# Permeation Damage of Polymer Liner in Oil and Gas Pipelines: A Review

**DOI:** 10.3390/polym12102307

**Published:** 2020-10-09

**Authors:** Hafiz Usman Khalid, Mokhtar Che Ismail, Norlin Nosbi

**Affiliations:** Department of Mechanical Engineering, Universiti Teknologi PETRONAS, Perak 32610, Malaysia; mokhtis@utp.edu.my (M.C.I.); norlin.nosbi@utp.edu.my (N.N.)

**Keywords:** NMP, permeation, pipeline failure, coupons, dielectric

## Abstract

Non-metallic pipe (NMP) materials are used as an internal lining and standalone pipes in the oil and gas industry, constituting an emerging corrosion strategy. The NMP materials are inherently susceptible to gradual damage due to creep, fatigue, permeation, processing defects, and installation blunder. In the presence of acid gases (CO_2_, H_2_S), and hydrocarbons under high pressure and temperature, the main damage is due to permeation. The monitoring of possible damage due to permeation is not well defined, which leads to uncertainty in asset integrity management. Assessment of permeation damage is currently performed through mechanical, thermal, chemical, and structural properties, employing Tensile Test, Differential Scanning Calorimetry (DSC), Fourier-transform Infrared Spectroscopy (FTIR), and Scanning Electron Microscopy (SEM)/Transmission Electron Microscopy (TEM), to evaluate the change in tensile strength, elongation, weight loss or gain, crystallinity, chemical properties, and molecular structure. Coupons are commonly used to analyze the degradation of polymers. They are point sensors and did not give real-time information. Polymers are dielectric materials, and this dielectric property can be studied using Impedance Analyzer and Dielectric Spectroscopy. This review presents a brief status report on the failure of polymer liners in pipelines due to the exposure of acid gases, hydrocarbons, and other contaminants. Permeation, liner failures, the importance of monitoring, and new exclusive (dielectric) property are briefly discussed. An inclusive perspective is provided, showing the challenges associated with the monitoring of the polymer liner material in the pipeline as it relates to the life-time prediction requirement.

## 1. Introduction

Pipelines play a substantial and dynamic role in the transportation of crude oil and natural gas. Crude oil and natural gas contain various corrosive contaminants such as CO_2,_ H_2_S, water, and microbes. CO_2_ corrosion (sweet corrosion) and H_2_S corrosion (sour corrosion) are the most prevalent forms of corrosion [1,2]. The rate of internal corrosion in wells and pipelines is influenced by CO_2_ and H_2_S content, water, flow velocity, and surface condition of the material.

Internal pipeline corrosion can be mitigated by several means, including the selection of appropriate material, use of corrosion inhibitors, metallic and non-metallic linings, or coatings [3]. The practice of using non-metallic pipe (NMP) materials in oil and gas production and transportation is emerging as a viable and reliable solution to mitigate corrosion. Polymers and composites both fall under the category of NMP, but we only discuss polymers in this article and specifically thermoplastics. NMP materials have applications in both onshore and offshore pipelines [4]. NMP has the advantage of low weight and better corrosion resistance as compared to metals. However, the possibility of permeation under high temperature-pressure, hydrocarbons, and acidic gases limits the application [5]. Thus, the systematic evaluation of possible degradation mechanisms and phases is critical for the successful use of NMP materials.

Permeation is a slow process and requires sensitive monitoring methods. Currently, test coupons are used for monitoring NMP degradation. These coupons were inserted in the pipelines, and after some specific time intervals, they are retrieved and evaluated based on using molecular weight as a measuring indicator. The coupons method only provides qualitative measuring for degradation mechanism, but no real time information is possible, therefore it needs to be further explored in terms of quantitative measures.

### 1.1. Corrosion in Oil and Gas Pipelines

The various parts that are susceptible to internal corrosion are downhole tubing, surface pipelines, pressure vessels, and storage tanks. This internal corrosion could lead to catastrophic failure causing severe consequences to the population, assets, and environment [6,7]. Seventy percent of pipeline failures in the oil and gas industry are due to corrosion, and 58% of these occurred internally [8]. Internal corrosion in pipes is influenced by temperature, corrosive gases (CO_2_ and H_2_S), water chemistry, flow velocity, oil or water wetting and composition, sulfate reducing bacteria, and surface condition of the pipe material. Any change in these parameters could influence the corrosion rate significantly because it would alter the properties of the corrosion products that form on the metal surface. Corrosion is present in various forms in the oil and gas production, which are sweet corrosion, sour corrosion, oxygen corrosion, galvanic corrosion, crevice corrosion, erosion-corrosion, microbiologically induced corrosion, and stress corrosion cracking [9].

Carbon dioxide (CO_2_) is a principal corroding agent and a recognized problem in the oil and gas production facilities. The partial pressure of CO_2_, water chemistry, pH, and temperature are the prominent parameters affecting sweet corrosion. Sweet corrosion was first recorded in the 1940s in the US oil and gas industries [10,11]. Sweet Corrosion covers almost 60% of failures in the oil and gas pipelines. The water itself is not corrosive but plays a significant role in making the environment acidic by reacting with CO_2_ and H_2_S [12,13]. Dissolved carbon dioxide in the produced or condensed water can result in a very high corrosion rate. Lack of corrosion management is the other contributing factor in the failures of the company’s assets.

### 1.2. Mitigation Practices

The integrity of the pipeline is critical; therefore, sufficient corrosion management should be implemented to optimize production. CAPP (Canadian Association of Petroleum Producers) has developed several industry practices for reducing the risk of internal corrosion incidents due to sweet and sour environments [14,15]. The mitigation can be achieved by specific measures such as the selection of appropriate material, coatings, corrosion inhibitors, and internal linings [16].

The principles of corrosion need to be understood to select the proper material [17]. Carbon Steel (CS) is frequently used, but its practice was limited due to reduced corrosion resistance in the oil and gas environment [11]. CS, in combination with continuously injected corrosion inhibitors, is occasionally feasible and cost-effective [18]. On the other hand, corrosion-resistant alloys (CRA) are considered to be the best option, but their usage drops with time due to high capital cost. There is a wide range of CRA fall under the category of Stainless Steel (SS) for the oil and gas pipelines [19].

Coatings are used to create a barrier between the working pipeline and fluid media. It can be metallic and non-metallic [17]. Sometimes the coating is not defect free, so the deployment of corrosion inhibitors is necessary to prolong the life of pipeline [20]. Corrosion Inhibitors can mitigate corrosion by two mechanisms; one is to control the chemical composition of the environment and the second one is by adsorbing on the metallic wall or surface and form a protective barrier. They are not a feasible option for high temperature operating conditions. They are classified according to their influence on the corrosion reaction, type of metal used, and the environment. Moreover, the effectiveness of corrosion inhibitors depends on the fluid composition, quantity of water, and flow regime [16,21,22]. Table 1 sheds some light on the corrosion mitigation strategies in the oil and gas environment [9].

The emerging alternative is NMP for combating internal pipeline corrosion. These liners are preferred as the most feasible corrosion management solution in cases where the long-term reliability of chemical corrosion inhibition systems is not suitable, and sometimes the inhibitor consumption rate is so high that it becomes more expensive than a liner over the lifetime of the pipeline. NMP materials are considered to be one of the best available and most feasible measures to prevent pipeline integrity.

## 2. Use of Non-Metallic Pipe (NMP)

Non-metallic pipe materials are comprised of both polymers and composites, and they have been used as an internal liner for many years. In the case of internally corroded pipes, the use of thermoplastic liners for rehabilitation is a feasible option to extend the lifetime of pipelines and reduce maintenance costs. This relining technique was first used in Europe and North America [23]. They have many unique properties comprising lightweight, corrosion-resistant, chemical inertness, and excellent resistance to heat and combustion. Thermoplastic lined pipes proved to be commercially applicable in one of the reports given by Atkins Boreas [24]. Thermoplastic liners (TPL) were also used to control downhole failures in oil and gas production as compared to other mitigation strategies, including coatings and chemical treatment. The usage of TPL was cost-effective, as proved by the case studies in Canada, Basin, and Bahrain [25]. They provide a competitive advantage in terms of cost and lifespan, compared with CRAs.

In thermoplastics, High-density polyethylene (HDPE), polyamide (PA11, PA12), and polyvinylidene fluoride (PVDF) are the most common polymer material used in the pipeline industry. NMP material composites divide into two groups FRP (Fiber-reinforced plastic) and FRE (Fiber-reinforced epoxy). The matrix can be made from thermoplastic, thermosetting, and elastomeric materials, and the reinforcement comes from glass, aramid, and carbon. The composites are used for the downhole and upstream applications. These raw materials used to make the final composite pipe such as reinforced thermosetting resin (RTR)pipe, reinforced thermoplastic pipe (RTP), and the most recent technology thermoplastic composite pipe (TCP) [26].

Multi-layer foil composite (MLFC) performs better for corrosion protection as compared to most thermoplastics including LDPE, HDPE, and nylon. Corrosion protection of steel pipelines with metal-polymer composite barrier liners [27]. The pipelines equipment’s of the ships are made from Polymer composite material (PCM) with several properties including higher strength to mass ratio, chemical inertness, low thermal conductivity, and better protection against electrochemical corrosion. Three-layer PCM pipelines provide the most enhanced shield against the flowing gases (CO, CO_2_) in the pipelines at higher temperatures. The inner shell provides rigidity and reduces the environmental temperature effect that improves the pipeline’s life [28].

**Figure 1:**
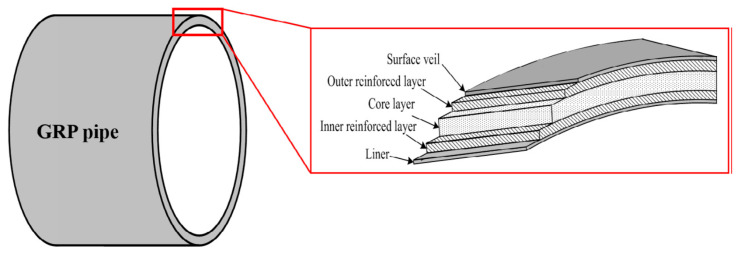
GRP Pipe [30].

**Figure 2:**
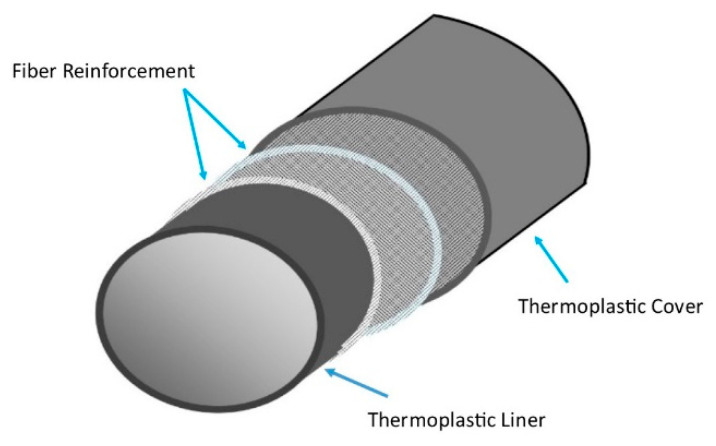
RTP structural components adapted from [31].

Thermoplastics liners follow the NACE RP-0304 standard 2nd edition for their design, installation, and operation in the oilfield pipelines. ISO 23936-1:2009 provides a summary regarding the performance of different thermoplastics with interaction with produced water, oil, and gas media along with chemical treatment [29]. Table 2 shows the usage of different NMP materials in the oil and gas pipeline applications. Figure 1 shows the different layers present inside the glass reinforced plastic (GRP) pipe while Figure 2 shows the internal and outer structure RTP pipe.

### 2.1. Flexible Pipes

Flexible pipes are used due to their flexibility and strength in offshore production and transportation systems. Non-metallic pipes, including reinforced thermoplastic pipe (RTP), thermoplastic composite pipe (TCP), and flexible composite pipe (FCP), are introduced as a substitute to CS rigid pipelines. The internal and external layers are made of thermoplastic materials, while the reinforcement consists of glass fibers, aramid fiber, GRE, and steel cords [51,52]. NMP materials can be used in many applications in the form of reinforced thermoplastic pipe, rigid and flexible risers [32]. Flexible risers are used in the deep sea and shallow wells worldwide. Flexible pipes play a crucial role in the deep-water offshore exploration and production systems. They are classified into the bonded and unbonded structure. In bonded, the reinforcement is embedded in polymer while in the unbonded single polymeric pipe is used besides steel cover [53,54]. As a result of swelling and blistering, bonded pipes cannot be reliable in the presence of gas and crude oil mixtures. The inner polymeric sheath also is known as a pressure barrier that works as a sealing, insulating, and anti-wear component. At the same time, the metallic layer withstands the structural load and prevents collapse [55].

Infield liner (IFL) consists of outer thermoplastic polyurethane, middle aramid core and the internal layer made of PVDF. It is used for rehabilitation of offshore pipelines. Two IFLs installation were carried in offshore pipelines in Malaysia, no gas permeation was observed due to highly impermeable properties of PVDF [42]. Kevlar reinforced polymer composite liner also known as IFL increased the burst pressure of the corroded host pipe. The reason for enhanced burst pressure is that the fabric stretched inside the defect cavity and induced the load transfer [56].

PA11 was employed as an internal insulation layer in flexible pipelines because of its excellent properties and chemical resistance during the crude oil and gas transportation. The only drawback aspect is its sensitivity to the hydrolysis phenomenon that will induce morphology changes and the chain scission. The hydrolysis is accelerated by high temperatures and low pH conditions [57,58].

**Figure 3:**
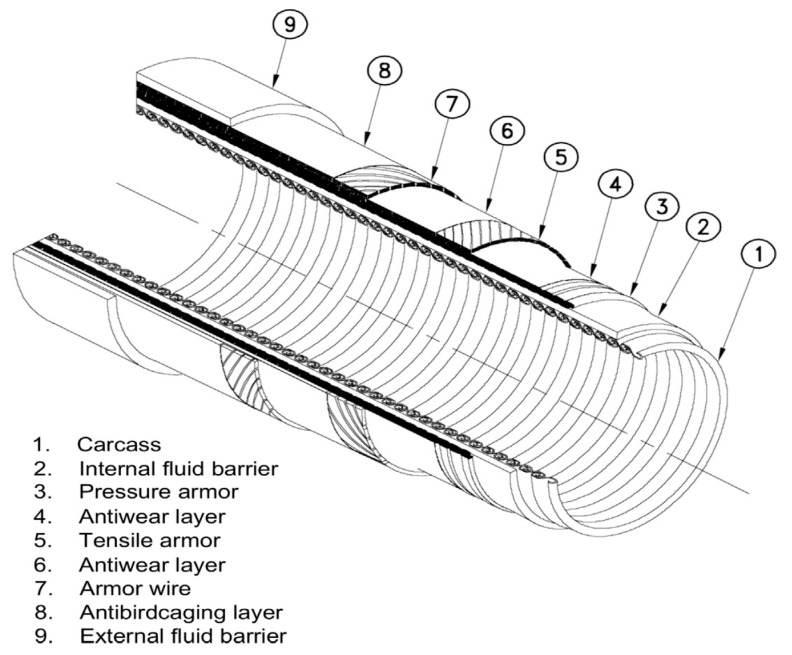
Flexible pipe [64].

PE and PVDF are also used depending on their compatibility and chemical resistance. PE-100 liner was installed in 6” and 16” CS subsea pipelines by swagelining technique, its performance checked by bending and pressure testing. The liner showed the less flexural modulus and no rupture was seen during pressure testing [59]. PVDF is a viscoelastic material, and it gets thinner rather than producing a neck when tension is applied, one of the reasons for using it in flexible pipes [60]. PA has been used for 30 years in the flexible pipes due to its ability to bear the mechanical load in dynamic applications [61,62]. Plasticizers are added to improve the elasticity and reduced the strength. PA11 and PA12 can be used within the temperature range of 100 °C for the transportation of crude oil. However, Coflon (PVDF) will be used for higher temperatures and HDPE for hydrolytic resistance [63]. Figure 3 shows the layered structure of the flexible pipe.

### 2.2. Degradation of NMP Material

Failures of non-metallic equipment in oil and gas production can occur due to multiple causes. Table A1 in Appendix A compiles different failure types of NMP material in the oil and gas industries. Degradation of polymers occurs due to permeation, absorption, oxidation, and hydrolysis took place in the pipelines [65].

**Figure 4:**
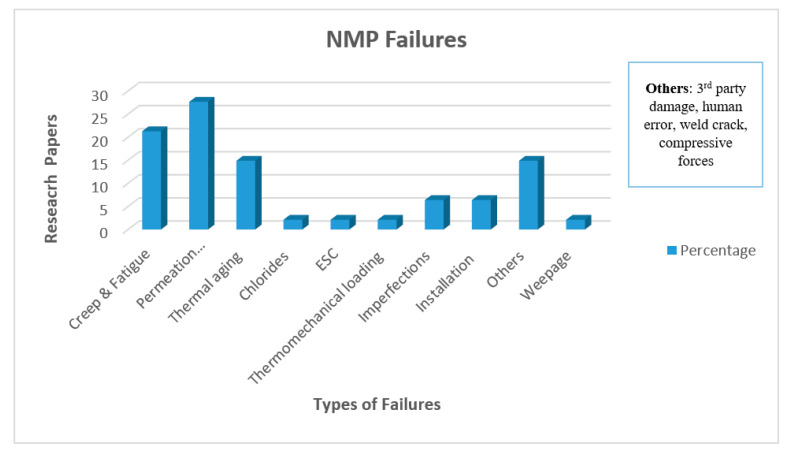
Failures in NMP material.

The failures occurring in the NMP materials were demonstrated in Table A1, while the frequency of failures is shown in Figure 4:

As we see from Figure 4, there are many failures adopted by NMP. Still, permeation is the prominent one, and it was studied by various researchers up until now in different environments on different materials. Permeation being the dominating factor in the polymer liner failure. In the presence of hydrocarbons, polyolefins swell as they both have a similar chemical structure. Polyamide degrades due to the presence of water at higher temperatures [66].

#### 2.2.1. Permeation

Permeation is a molecular phenomenon, involving the passage of a fluid, gas, or vapor through a material. It is a naturally occurring phenomenon, so it is a very much vital process to focus on the subject matter. Permeation is a function of two variables:

1. Diffusion (D) between molecular chains
2. Solubility (s) of the permeant in the polymer

The driving force for diffusion is the partial pressure of gases and the concentration gradient of liquids [67,68,69]. However, the diffusion mechanism also often improves by the chemical potential gradient [70]. It is the kinetic parameter which reflects the mobility of the penetrant in the polymer structure. Solubility is a thermodynamic parameter that shows the affinity of the permeant for the polymer. First, adsorption of permeate onto the polymer surface then diffusion from a higher concentration to lower concentration. Permeation and degradation are related up to some extent; for instance, the components of the permeation process (solvation and diffusion) give hostile chemicals a route into the polymer bulk. Fick’s law of diffusion best describes permeation models. It provides the following Equation.

Q = Ds(1)

where Q(Pe) is permeation coefficient, D is the diffusion coefficient, and s is the solubility coefficient.

Thermal expansion and hydrocarbon swelling will generate stresses in the liner material, which leads to buckling or collapse of the liner. Thermoplastics give a free path for gases, vapors, and water to pass through them. Barrer was the first one to explain that the diffusion of molecules through rubbery polymer is a thermally initiated process [71]. Permeation mainly depends on the number of critical factors, including liner thickness, partial pressure, % crystallinity, crosslinking, size of the permeant, and the gap between the liner and the host steel pipe [72]. The presence of a higher degree of crystallinity lowers the diffusion and solubility in PE [73]. The process of permeation is primarily a function of temperature. Temperature affects gas permeation prominently [74,75]. Diffusion and permeation parameters are temperature-dependent. They increased by increasing the temperature while solubility had an opposite relation with it. Henry gave the law of absorption, which explained the direct relationship between solubility and pressure. It is not always linear, which was stated by Langmuir, BET, and dual sorption models that the permeation coefficient may or may not vary with pressure [76].

Permeation through NMP materials is a distinct aspect of the degradation of pipelines. It happens due to the exposure of acidic gases and immersion in hydrocarbons, including acids in the presence of temperature and pressure. Table A2 sheds some light on the permeation and absorption of gases and liquids present in the oil and gas pipelines through different types of NMP materials.

**Figure 5:**
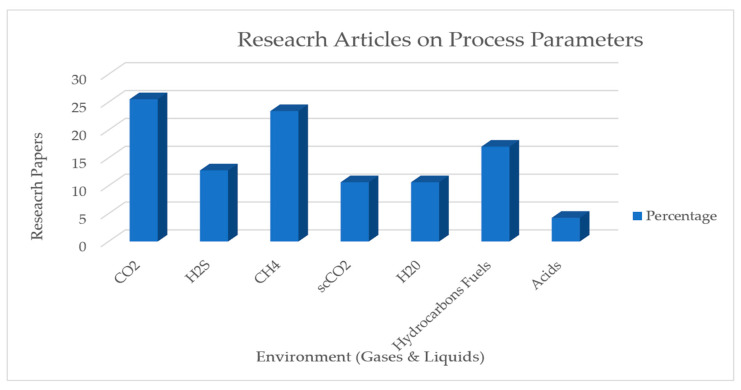
Different types of gases and fluids used in permeation experiments.

We can conclude from Table A2 that how different gases present in the environment of the pipeline affect the liner materials. Figure 5 shows the study of various gases in permeation from 1996–2020.

**Figure 6:**
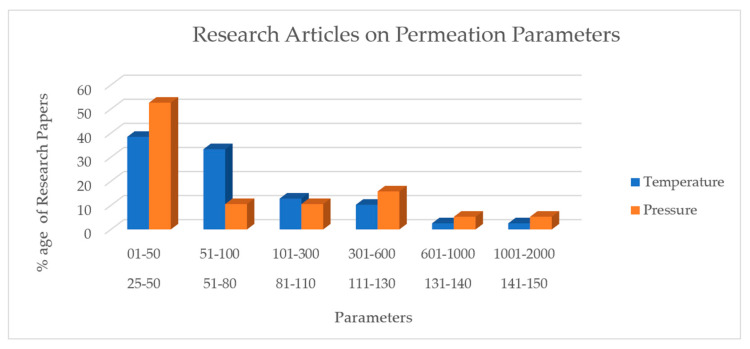
Process Parameters in Permeation Experiment.

Temperature and pressure also play an important role in the permeation phenomenon. The different polymeric material behavior was assessed using these parameters. Figure 6 shows the percentage of research articles published on these parameters within the previous years from 1996–2020.

**Figure 7:**
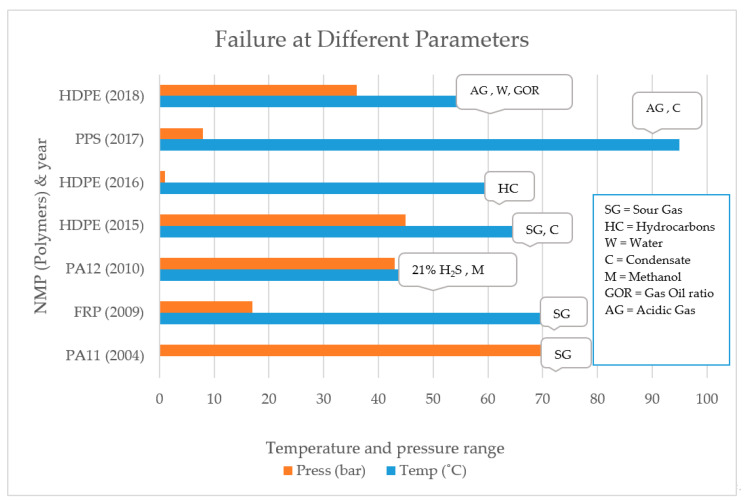
Polymer Liner failures in previous years.

Failures of polymer liners in the previous years are shown in Figure 7.

#### 2.2.2. Flexible Pipelines

Permeation is a vital parameter to consider during the lifetime of flexible pipes. The vents are most commonly present on the connection end fittings. It follows two problems, one that the permeate gases most likely to corrode the reinforcements. The other one is that the water condenses in the annulus and restrict the flow of gases. Due to this phenomenon, the buildup of pressure occurs, which in turn causes the outer sheath to burst. Some state-of-the-art solutions were given in U.S Patent. The removal of permeate gases was carried out by injecting inert gas (N), which forces them along the venting path; another one is to use a suction pump [77]. PVDF is susceptible to water permeation. It was proved by a large-scale test at 100 °C and 50 bar in the presence of CH_4_ and CO_2_ gases. The permeation of CO_2_ is more than CH_4_ [78]. IFP and Technip provided a solution to obstruct H_2_S permeation. Anti-H_2_S layer containing PEZnO was placed between the pressure sheath and annulus to its effect. The reaction between oxide and this anti-H_2_S layer gave a purple color, which was observed by the optical microscopy and EPMA [79]. Due to a higher percentage of ethanol content (85%) and temperature, swelling occurred, which in turn increased the flux across the PA12 multi-layer pipe. The delamination of the inner PA12 layer from PVDF due to the presence of ethanol fuel. The loss of plasticizer at higher temperatures affects the performance of the pipeline [80]. Hydrolysis is the central primary damage for PA in flexible pipes include the breaking of amide linkages in the backbone, reducing the molecular weight. Three dog bone specimens were taken from three different layers of the flexible pipe operated at 80 °C for three years. The aging of PA is monitored by the solution viscosity (CIV-corrected inherent viscosity) value, and it decreased due to chain scission. Plasticizer loss happened due to high temperature service. The elastic modulus decreases with an increase in CIV value. The degree of crystallinity increased due to the phenomenon of chemi-crystallization [81]. The durability of the aliphatic polyamides with long alkyl chains such as PA11 and PA12 gave higher strength in the severe environment to inner sheath failure. The plasticizer loss is also a critical factor in materials embrittlement. Fracture and tensile tests were carried out at different temperatures, and CIV values were measured. No necking appears during the tensile test, and the deformation is homogenous. PA showed a brittle behavior at lower values of CIV and vice versa [55]. HDPE is a hydrophobic material, but susceptible to aromatic hydrocarbons and cyclic solvents. It is appropriate for use in hydrocarbons within the temperature range of 45 °C to 60 °C. However, in the presence of hydrocarbons, they tend to swell and lose their strength [5,82].

**Figure 8:**
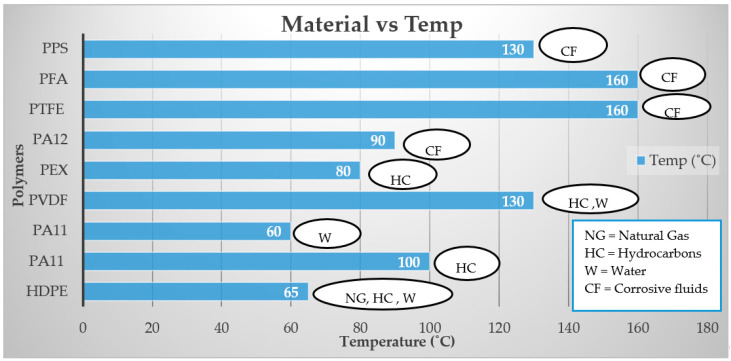
Temperature limits for Polymers.

Now we know that different polymers are degraded in different specific environments. Figure 8 shows the temperature limits for using these polymer materials in the oil and gas industry in diverse environments.

### 2.3. Thermodynamics of scCO_2_ (Supercritical) in Permeation

The interaction of CO_2_ with different polymers gave alternative prospects in their properties. CO_2_ is the most studied gas in the permeation process, as seen in Table A2. Therefore, in this section, we discuss what will happen when scCO_2_ comes in contact with different polymeric materials. A supercritical fluid is a fluid that has a temperature and pressure above its critical values, i.e., in the critical region. The density of supercritical fluid near a critical point is very sensitive to small pressure change. Cross-linked polyethylene (XLPE) and PVDF are the most influential polymers for high-pressure CO_2_ applications [83]. The contact triggered the swelling and plasticization of the polymer, which in turn reduced the mechanical properties and increased the permeation rate. There is a chance of blistering in terms of decompression.

In Deepwater offshore, the pipelines face a more chemically aggressive environment, including supercritical CO_2_ (scCO_2_). Unfortunately, they degrade with time. Experiments were run to predict the life of PA12 in the presence of crude oil with water and scCO_2_. The respected material did not perform well in the scCO_2_ environment and degrade earlier as compared to other environments [84]. The permeation was carried out at 45, 60, 75, 90 °C and 100 bars. The solubility increases with increment in pressure but decreases with higher temperatures for both polymers. Due to proximity to the critical point, the behavior of solubility was quite unusual at 100 bars [83].

PVDF was used due to its high temperature bearing capacity in the offshore risers. The effect of scCO_2_ on PVDF was evaluated. The high-pressure CO_2_ has a synergetic effect on the material and equipment. The increase in volume (24.17%) occur, followed by a decrease in density (12.5%), and the sample color changed. The reduction in mechanical properties was observed, including decrement in hardness and elastic modulus. The fracture surface showed the ductile behavior of the sample due to the softening of gas [85].

For downhole applications, the pressure keeps on rising, and the material needs to have higher mechanical strength and corrosive resistance. The performance of HDPE, XLPE, and PA11 was evaluated in the presence of supercritical CO_2_ fluid. The operating temperature and pressure were 95 °C and 241 bars. The properties, including yield strength, decreased, and % elongation increased for HDPE and PA, but no change observed for XLPE [86]. PVDF and MDPE were tested for permeation in scCO_2_ at 77 °C and within a pressure range from 1–1000 bars. Well, other cases reported the coefficient of thermal expansion was reduced in the presence of supercritical fluid (CO_2_). As soon as the pressure increases, the sorption kinetics improved, and the fluids penetrate through the interstitial sites of the amorphous phase, degraded the mechanical properties [87].

The permeation experiment was carried out with MDPE and PVDF at 118 °C and 430 bars in the presence of scCO_2_. The swelling did not affect solubility around 100 bars, but above it, the change in volume was significant. The derived equation showed the key importance of gas density with its solubility as well as the geometry of the sample did not affect the solubility as long as the system is in equilibrium [88]. NKT flexibles have carried out an extensive qualification program for polymer liners in high-pressure CO_2_ applications. XLPE liner passed the blistering and high decompression test in the presence of 90 °C/650 bars CO_2_ due to its crosslinking effect in structure [89].

## 3. Inspection and Monitoring Techniques

Non-metallic materials are increasingly used in the oil and gas industry due to their distinguishable features such as corrosion-resistant, lightweight, ease of installation, and low maintenance costs. Like their metal counterparts, they have certain limitations such as chemical degradation, the impact of aging, and constraints on inspections. Inspection and monitoring of non-metallic pipes using in the oil and gas pipelines need to be done at regular intervals. Monitoring gives you an early indication of the damage which is going to influence your asset in the long run. Inspection measures the actual extent of the damage done. The degradation of NMP materials in the oil and gas pipelines modifies mechanical, thermal, and structural properties. The analysis of these properties is made by various characterizing techniques, including the tensile test, DSC, FTIR, and SEM/TEM. These techniques are used for testing the polymer’s behavior after a particular degradation process. However, we need to monitor it before it can proceed to the failure of polymer materials. It can be seen from the literature that after degradation in different environments, polymer properties demonstrated a significant change. To exhibit the performance of polymers, they are aged in the lab before use in the field. Table 3 shows the degradation of certain polymers in specific environments and the methods used for their characterization.

Cracking appears in the HDPE specimen when the temperature exceeds 80 °C, and the % elongation is reduced following the increase in temperature [90]. The thickness comparison was carried out as the minimum thickness polymer showed faster aging and embrittlement due to the formation of surface cracks. The simultaneous reduction of elongation at break and oxidative onset temperature (OOT) indicate the reaction of ClO_2_ with AO (antioxidants) and polymer molecules. Various peaks were observed in the FTIR curve, indicating phenolic AO degradation [91]. After induced aging in hot air and deionized water, PE-RT and PP-R showed higher enthalpy values. Due to the temperature increase, the strain at break decreases [92]. PVDF presents low hardness and elastic modulus values, in the presence of crude oil. Plasticization occurs due to the swelling of polymer material [93].

The embrittlement of both PE grade materials was demonstrated by an increase in the carbonyl index value. Degradation of mechanical properties is attributed to the reduction of molar weight caused by the β-scission of alkoxy radicals [96]. Diffusion and degradation of low molecular weight chains occur in HDPE due to aging in the presence of diesel lubricants. No chemical modification in the structure was observed [94]. Mechanical properties and the crystalline structure were unaffected after one year of exposure to hydrogen gas [97]. Oligomers were formed by the hydrolysis of PA11 in the polymer matrix. The degree of crystallinity rises with respect to aging. CIV values tend to be lower at higher temperatures. Due to immersion in the oilfield water (pH = 5.5) for 50 days, surface cracks emerge in the SEM images [57]. Structural properties and shore hardness were measured after exposure of TM mixture to PE. The surface roughness of the internal surface increased after 1290 days. Structural changes occurred due to aging in the presence of TM mixture and properties, including % age crystallinity and thermal stability of material decreased. The value of Young’s modulus significantly dropped, and the pipe shifted toward brittle behavior [95].

### 3.1. Coupons

Coupons are used because of their simple working principle and smooth operation. However, installation, removal, and lab analysis require an extended period. Inline monitoring using mounted test coupons is one of the techniques to test polymer aging. It does not represent the most critical bore environment, so it is not that feasible. Installing coupons on remote, buried pipelines is often impractical at susceptible locations. They are point sensors with limited sensing coverage and did not provide real-time information on oil and gas infrastructures. Force Technology, one of the leading companies, implicates the monitoring of flexible risers using vent gas monitoring, polymer coupon monitoring, and load and response monitoring. Coupons are used to monitor the integrity of the polymer sheath layer within the flexible risers. They are placed in the pipeline to be exposed to the same conditions. Coupons are retrieved for monitoring and examined with their patented method for evaluating the integrity of the polymer sheath [98]. The dielectric sensing technique for the degradation of polymer uses test coupons placed inside the pipe bore rather than test coupon removal, but it is under development now [99].

**Figure 9:**
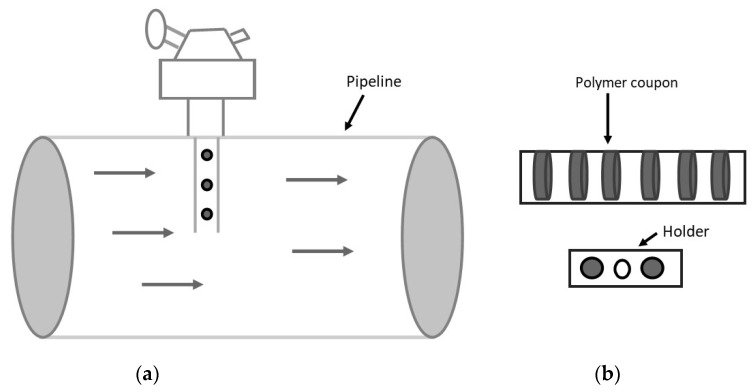
(**a**) Polymer coupon in the pipeline, (**b**) coupon and holder.

Pressure sheaths of the production flexible pipes on the Atlanta field are made of PA12 material. To ensure the mechanical integrity of the pipeline, polymer coupons were installed for monitoring the condition of the pressure sheath. The coupons were removed regularly, and the molecular weight is their measuring indicator. The analysis to assess deterioration will follow the methodology outlined in the API 17TR2 [100]. It was previously briefed in one of the U.S. Patents to predict life and monitor the changes in the installed coupons. Coupons were tested in the lab by accelerating the parameters to achieve physical and chemical aging. However, before running the experiment, these pre-aged coupons were evaluated for different properties, including tensile strength, modulus, elasticity, and crack initiation and propagation [101]. Before the testing of grooved liners in the pipelines, coupons were placed to verify the performance of the material [86]. One of the fields in Canada had a project of installing PA12 liner. Before the installation, coupons were installed for one year. Mechanical properties and molecular weight were determined after the retrieval [102]. For predicting the life of pressure sheath in a flexible pipe, the aging of Rilsan (PA11) was monitored by placing the coupons inside the flowline. Coupons are served as an integrity management tool [103]. Due to degradation mechanisms such as hydrolysis and oxidation, polymer chain scission occurs, which gives shorter segments of chain. It will affect the elongation at break value suddenly. One novel method was developed to measure the mass fraction of polymer. PA11 material was separated into individual chains. These chains were separated by size and the size is measured by refractive index or UV absorption technique. Then these molecular weights can be analyzed by the computer [104]. Figure 9a,b shows the disk shape polymer coupon and its insertion in the pipeline.

### 3.2. Dielectrics

A dielectric material is a substance that is a poor conductor of electricity or an insulator but can be polarized by the electrostatic field. Polymers are dielectric materials, and they can be electrically polarized by applying an external field. Polar and non-polar groups characterize them. Dielectric materials are solids, including ceramic, glass, and plastics. The extent to which the material can concentrate the electrostatic lines of flux is dielectric constant. If the voltage is high, the field becomes too intense, and the material is damaged permanently. Important parameters describing polymers dielectric behavior are dielectric constant and dissipation factor. Dielectrics provide impedance to the flowing AC current, impedance includes both the resistive and the reactance components [105]. Different techniques are present to find dielectric properties, including Impedance Analyzer and Dielectric Spectroscopy.

Dielectric spectroscopy is used to measure the permittivity or dielectric constant of the polymeric materials as a function of frequency [106,107]. It can also be done by as a function of time or temperature at fixed frequencies to determine the physical and chemical properties of polymer. It also gives fundamental understanding of the molecular dynamics processes in polymers. Dipole reorientation (permanent and induced) and conduction of ions or electrons in the present of electric field contribute to the dielectric response of the material.

The permeation fluxes can be found using the Flory–Huggins and Maxwell–Stefan models. Both models describe the thermodynamics and equilibrium of the polymer solution [70]. These models are gaining popularity with time. It is one of the methods used to find the permeation across polymeric materials.

## 4. Conclusions

Carbon Steel pipelines are prone to corrosion attacks. NMP plays a promising role as a liner to prevent the pipelines from deteriorating due to their proficient features such as their light weight, low cost, ease of installation, chemically, and thermal inertness. Polymer pipes do not have a prolonged life due to permeation damage. They have an inherent nature to provide pathways to hydrocarbons and gases to permeate through them. Plasticization and swelling occurred as a result of hydrocarbon absorption. Certain important characteristics, such as tensile strength and elastic modulus decline.

Many other scholars explored the permeation of acid gases and hydrocarbons over the years. Its monitoring is crucial for the safety and integrity of the company’s assets. One common approach is the use and assessment of coupons in the field research. The molecular weight and mechanical properties act as a predictive approach to polymer degradation. It did not explain the process of damage, neither when it began nor how it continued.

## 5. Future Suggestions

The usage of coupons is more of a conventional approach; online monitoring is required if we are to find the premature failure in the polymer liner in oil and gas pipelines. As far as future prospects are concerned, the dielectric property is of the utmost importance. It may be used as an alternative means of monitoring the damage mechanism. For this purpose, experimental work will be required, impedance can be determined using different methods, and this value can be correlated with the dielectric property. It may be useful for quantitative measures to identify the degradation stages in the material after permeation damage. The outcomes of the dielectric property contribute to a more comprehensive and systematic post permeation monitoring approach. In this way, we can establish some acceptance requirements for the remaining polymer life.

## Figures and Tables

**Table 1:** Corrosion prevention measures [15].

Preventive Measures	Selection	Remarks
Material Selection	(a) Carbon Steel, Corrosion-Resistant Alloy (CRA) (b) Non-metallic materials such as thermoplastic lined polyethylene pipes	(1) Use of non-metallic material pipe as an internal lining. (2) Use of standalone pipe depending on the service condition.
Chemical treatment	(a) Corrosion inhibitors (b) Oxygen scavengers (c) Biocides	(1). Provide a barrier between the pipeline and fluid. (2). The presence of acidic gases, oxygen, and bacteria will accelerate the corrosion.
Coatings	(a) Organic and Inorganic (b) Metallic	(1). They are used for internal and external protection.
Process Control	(a) Categorize the crucial parameters: pH, temperature, pressure, flow rate, chlorides, bacteria, oxygen, and acidic gases	(1). Manipulate the operating conditions will play a role in mitigation. (2). Dissolved metal concentration (i.e., Fe, Mn) specify the changes in the corrosion phenomenon.

**Table 2:** NMP applications.

Material	Properties
HDPE	It made a significant proportion of oil and gas pipelines [32]. With valuable properties such as semi-crystallinity, high strength, and low-density [32,33,34]. They have a smoother pipe surface with low thermal conductivity [14]. PE100 and PE4710 are used for pressure-rated applications following ISO and American Society for Testing and Materials (ASTM) standards. The grooved liner is used for rehabilitation of CS aged pipelines [35]. No swelling or softening was observed in PE installed liner in Casabe’s field located in Colombia after 12 months of service [36].
PA	It is a synthetic polymer, also known as nylon. It has a semi-crystalline structure with an amide linkage between monomer groups. It was used where HDPE is not applicable in terms of high temperature [37]. PA11 and PA12 have better hydrolytic resistance as compared to Nylon 6 and Nylon 66 [38]. PA12 used a cost-effective solution as compared to CRA and cladding. Its liner is suitable for hydrocarbon services for temperatures above 55 °C [39]. No change in the molecular weight of PA11was observed except for some plasticizer loss after two months in sour production pipeline in Canada [40]. BP Amoco installed PA11 liners in one of their hydrocarbon production lines operated at 60 °C with 17% H_2_S due to its excellent chemical and abrasion resistance.
PVDF	Due to the presence of C-F bonds, it has a high melting point (177 °C) and excellent mechanical properties, which gives powerful resistance to the fluids producing in the oil and gas environments. With exceptional features, including good abrasion and chemical resistance, low coefficient of friction, and low moisture absorption [41]. Its mechanical and thermal stability with a working temperature limit of 130 °C makes it suitable for liner applications [42]. Recommended using in the presence of petroleum products, O_2_, CO_2,_ and chlorinated water within the temperature range of 110–125 °C. It is not affected in the presence of alcohol, chlorinated solvents, aromatic and aliphatic hydrocarbons [43]. PVDF-PLP can be used in acidic service conditions for oil and gas flowlines, it is cost-effective than CRA and has better flow assurance properties [42]. PTFE and Perfluoroalkoxy alkane (PFA) can handle a variety of solvents, including acids, alkalis, and corrosive fluids up to a working temperature range of 160 °C [44].
GRE	GRE (Glass Reinforced Epoxy) has a high strength to weight ratio, easy installation, and a better life span [45]. They are used for extensively employed for transporting corrosive hydrocarbons [46,47].
FRP (GRP)	It is used due to its low weight and corrosion-resistant abilities. It can replace steel pipes in terms of operational cost [36] With a smooth surface which minimizes the scale formation and improves fluid hydraulics [48]. Used in WWII to replace glass fiber cloth for crude oil applications [49]. FRP pipes can be installed in a seamless manner upto many miles for hydrogen transmission [50].
RTP	Used for gas transportation in onshore and offshore pipelines. With exceptional properties such as high strength, flexibility, corrosion resistance, and ruggedness [26,36]. The bonded product gives suitable resistance to liner collapse or buckling due to permeation [32]. The commercial manufacturers of RTP are Airborne, Pipelife, Technip, Coflexlite, and Cosmoplast.

**Table 3:** Characterization of Polymers.

Research Article	Materials	Parameters	Time Interval	Characterization Techniques
Fu, et al. [90]	HDPE	CO_2_, H_2_S, O_2_ and TH 4XX formation water, 80, 110 and 140 °C 10MPa	562 days & 30 days	Tensile test
Bredács, et al. [91]	PE (2 grades)	ClO_2_ Immersion, 60 °C 1 ppm 5 & 10 ppm	One Week	SEM, Tensile test, FTIR, Dynamic Oxidation Test
Grabmann, et al. [92]	PE-RT PP-R	Hot Air & De-ionized water 115 °C, 50 °C, 95 °C & 135 °C, 115 °C, 95 °C	77 to 1372 days	Tensile Test, DSC
de Oliveira, et al. [93]	PVDF	Crude Oil 80 °C 1 atm	30, 120, 320 days	Instrument Indentation Technique
Torres, et al. [94]	HDPE	Diesel 20 °C & 50 °C	150 days	Thermogravimetric & FTIR
Romão, et al. [57]	PA11 (plasticized)	Deionized water, Oilfield water 110, 120 & 140 °C	30 & 50 days	DSC, SEM, XRD
Ghabeche, et al. [95]	HDPE	Toluene-methanol Ambient	7 & 1290 days	DSC, tensile test

## Appendix A

**Table A1:** Failures of NMP material.

Research Article	Damage	Remarks	Year
Learnings from thermoplastic liner failures in sour gas pipeline service and replacement liner design and installation [108]	Permeation (Exposure)	Permeation of sour gas (H_2_S) through the thin wall and loosely fitted liner caused buckling collapse.	2004
Analysis of ductile and brittle failures from creep rupture testing of high-density polyethylene (HDPE) pipes [109]	Creep	A majority of failures in pressure pipes were due to the SCG (slow crack growth). The transition from ductile to brittle failure is at higher test temperatures. The chemical knee shifted to shorter periods at lower stress and higher temperatures.	2005
Supercritical Gas–Polymer Interactions with Applications in the Petroleum Industry. Determination of Thermophysical Properties [110]	Permeation (Diffusion)	The effect of sc CO_2_, N_2_ on MDPE, and PVDF were carried out. The high concentration of gas in the polymer causes explosive deterioration in the form of blisters and cracks. CO_2_ sorption is favored in PVDF due to the presence of polar groups C-F.	2006
Oilfield Engineering with Polymers [111]	Thermal aging, Permeation	The author discussed the polymers applications along with design strategies and failure mechanisms. Perfluoro ether is better than Fluor elastomers in terms of temperature flexibility and resistance to acids and alkalis. Fluor elastomers are considered best for downhole applications.	2006
Failure analysis of polyethylene gas pipes [112]	Fatigue	The presence of residual stresses and variances in morphological changes affect the life of the HDPE pipe. The fluctuating operating pressure indicates fatigue loading.	2008
Effect of Fiber-Reinforcement Material on the Leakage Failure In Polymer Composite Pressure Piping [113]	Internal Pressure	The performance of fiber reinforcements for FRP was evaluated using E/ECR glass, Basalt, and S2 glass. Due to internal pressure and axial loading, cracks appeared, which caused leakage in the pipeline. The leakage strength improved by the S2 glass fiber.	2008
Creep damage mechanisms in gas pipes made of high-density polyethylene [114]	Creep, Fatigue	Creep damage behavior was investigated in an extruded HDPE pipe. Samples taken from different layers of pipe have different rates of creep. Fluctuation in pressure causes mechanical failure.	2009
Failure Types of FRP Pipe in Oil and Gas Engineering [115]	Thermal aging, Installation	The resin matrix is the weakest point in the FRP structure. In the presence of acidic gases and temperature, mechanical and thermal properties significantly decreased	2009
Corrosion failure in a lined sour gas pipeline—part 1: Case history of incident [116]	Permeation	One of the pipelines installed by Shell Canada Energy was failed after four years. Methanol permeates through the liner due to its high vapor pressure than water and caused failure.	2010
Effects of thermal aging on mechanical and thermal behaviors of linear low-density polyethylene pipe [117]	Thermal aging	The effect of temperature on the mechanical and physical properties of LLDPE after 6000h of aging was shown. An increase in the crystallinity and the crosslinking density; the pipe showed the brittle failure without plasticization due to thermally aging.	2010
Long-term Behavior of Polyethylene PE 80 Pressurized Pipes, in Presence of Longitudinal Simulated Imperfections [118]	Imperfection	The simulated imperfections were introduced to predict the service life of the PE-80 pipe. Ductile to brittle transition occurred, and the formation of cracks was seen.	2010
The Alberta experience with composite pipes in production environments [119]	Installation	Around 57.7% of leakages are due to internal corrosion. FRP and Spoolable Composite Pipe (SCP) were used in Canada to cater to corrosion. The rigid FRP faced the most frequent failures as compared to SCP and steel pipeline.	2010
Effects of ethanol content and temperature on the permeation of fuel through polyamide-12-based pipes [103]	Permeation	In the presence of higher ethanol content (85%) and temperature, swelling occurred. The internal delamination layer of PA12 occurred. Loss of plasticizer affected the pipeline performance	2010
Fatigue Analysis Of PE-100 Pipe Under Axial Loading [120]	Fatigue	Due to internal and pressure, stress develops in the circumferential, longitudinal, and radial direction. These stresses can produce cracks that result in the ultimate failure of the material.	2011
Non-Metallic Pipe Systems For Use In Oil And Gas [32]	Weepage	The fiberglass pipe showed weepage failure, and it occurred at lower pressures. The composite pipes showed time-dependent deformation in the form of matrix cracking.	2011
Application and Qualification of Reinforced Thermoplastic Pipes in Chinese Oilfields [121]	Installation External damage	The reinforced fiber got damaged at higher temperatures. In Changqing oilfield, RTP was damaged due to installation. Some of the pipelines failed due to external forces, including stones and construction equipment.	2011
Failure Analysis Of Steel Wire Reinforced Thermoplastics Composite Pipe [122]	ESC	25% of plastic part failures are due to ESC. The cracks are initiated from inside the pipe and move along radially. SEM analysis showed cracks and pores in the HDPE basal resin and the absorption effect by EDX.	2011
Fracture Surface Analysis in HDPE Pipe Material Fatigued at Different Temperatures and Loading Frequencies [123]	Fatigue	The material exhibited fatigue failure due to cyclic loading at different temperatures. Crack growth was dominant at the higher temperature (40 °C) while at 0 °C, only a limited amount of shear yielding occurred. The additive particles in the HDPE behaved as a stress concentrator and caused void formation.	2012
Analysis of a Failure in a Polyethylene Gas Pipe Caused by Squeeze off Resulting in an Explosion [124]	Compressive Stress (Brittle Fracture)	The PE pipe was squeezed for repair, but it produces the compressive forces on it. The stress concentrates on the inner surface of the pipe wall; covalent bonds broke down and caused the nucleation of brittle fracture.	2012
Numerical tool to model collapse of polymeric liners in pipelines [125]	Permeation	The organic components from crude oil caused physical swelling by destroying the intermolecular link. Acidic gases permeate through the liner wall and cause buckling collapse upon pressure fluctuation.	2012
Application of Non-metallic Composite Pipes in Oilfields in China [126]	Joint Failure, Condensate	The strength of polyester fiber reduced at higher temperatures around 65 °C as compared to aramid. SRTPs failed after one of the services due to temperature and external damage. Due to polymer flooding, the inner surface of GRP degraded while cracks appeared in the radial direction due to the presence of condensate.	2012
The effect of residual stress on polymer pipe lifetime [127]	Imperfection (Residual stresses)	The induction of residual stresses during the manufacturing process caused the formation of cracks. Residual stresses are very much important for the lifetime of the pipe structure.	2013
Advanced fiber-reinforced polymer (FRP) composites for the manufacture and rehabilitation of pipes and tanks in the oil and gas industry [128]	Permeation, Moisture	Glass fibers show a more permeable nature compared to carbon fibers. The moisture induction degrades the fiber/matrix interface, which reduced the stiffness. SCC also affects the lifetime durability of FRPC by decreasing its stiffness and modulus of elasticity.	2013
Multilayer polymer pipes failure assessment based on a fracture mechanics approach [129]	Creep	The crack was initiated due to the flowing pressure in the inner protective layer. In case of low adhesion between protective and main pipe, the crack propagates through the interface and vice versa.	2013
Failure analysis of anticorrosion plastic alloy composite pipe used for oilfield gathering and transportation [126]	Processing	The higher resin content and a lower degree of cure decrease the T_g,_ which affects the thermal stability and mechanical strength.	2013
Influence of specimen geometry on the slow crack growth testing of HDPE for pipe applications [130]	Creep	The SCG occurred at a low-stress level below the yield point. The formation of crack followed the crazing and micro-voids. The resistance to crack growth was studied by different geometries of PE sample using PENT and CDNT tests.	2015
Evaluation of Long-Term Behaviour of Polymers for Offshore Oil and Gas Applications [131]	Thermal aging	PA6 was thermally aged, and the decrease in Young’s modulus found out. The hydrostatic pressure increases the weight gain by the epoxy resins in the case of voids.	2015
Thermoplastic liners for oilfield pipelines [96]	Permeation	A four-inch CS pipeline installed by BP America was operated at 65˚C and 45bars. Due to the transportation of condensate, water, and acidic gas permeation phenomenon occurred. The pipe was inspected after six months and found out that it collapsed.	2015
Buckling collapse of HDPE liners: Experimental set-up and FEM simulations. Thin-Walled Structures [132]	Permeation	The absorption of hydrocarbons into thermoplastics causes swelling. The mechanical properties were reduced in terms of modulus and strength.	2016
Crack Damage in Polymers and Composites: A Review [133]	Fatigue	Impact and cyclic fatigue are the most common failures associated with polymers. In Fiber-Reinforced Composites, the failure occurs in terms of debonding of the fibre–matrix interface, matrix fracture, stress redistribution, fibre fracture, and fibre pullout.	2016
HDPE pipes failure analysis and damage modeling [134]	Fatigue	The fatigue requires three stages, initiation of microcrack, propagation, and rupture. Almost 50–90% of mechanical failures are due to fatigue. Notched specimens of HDPE tested, and their results were validated by damage models to predict the critical life fraction.	2016
Influence of aging in the failure pressure of a GFRP pipe used in oil industry [135]	Thermal aging	The GFRP pipes were aged using the temperature (80 °C) and pressure (1 MPa). The stiffness of the material is not affected, but the UTS is reduced by aging.	2016
“Failure of glass fiber-reinforced epoxy pipes in oil fields.” Handbook of Materials Failure Analysis with Case Studies from the Oil and Gas Industry [136]	Permeation (diffusion)	Diffusion caused the matrix plasticization, and swelling also reached the fiber-matrix interface. T_g_ decreased as well as mechanical properties degraded.	2016
Fracture and Mechanical Characteristics Degradation of Glass Fiber-Reinforced Petroleum Epoxy Pipes [137]	Chlorides	The GFRP were aged in petroleum water for 1440h. The presence of quartz particles in the epoxy resin allows the absorption of liquid chlorides. The fracture occurred, followed by the fiber breaking, pull out, and matrix breaking.	2016
Case Study: Engineered Polyamide 12 (PA12) Pipeline Liner for Management of Sour Gas Corrosion at Elevated Temperatures [102]	Permeation	HDPE liner failed after 24 months due to crude oil and high pressure in Lone Creek Field, Canada. PA12 was installed, and its performance checked after one year; permeation rates are not critical.	2017
Nonmetallics applications in oil and gas production (pipes, liners, rehabilitations), in Trends in Oil and Gas Corrosion Research and Technologies [5]	CO_2_ cracking, Thermal aging	A 3-inch RTP line with Polyphenylene sulfide (PPS) liner operating at 8 bar and 95 °C contains the produced fluid with high CO_2_ (47mol %), 5ppm H_2_S, and 97% water cut. It failed after two years in service due to cracking caused by the high CO_2_ content, high temperature, and the manufacturing anomalies acting as stress concentrators.	2017
Mechanical properties of offshoring polymer composite pipes at various temperatures [138]	Thermomechanical failure	Due to thermomechanical loading, degradation in softening point and T_g_ occur. Due to the increase in temperature, fiber matrix debonding, and delamination occurred.	2018
Study of Multilayered Composite Pipe subjected with Metal Interlayer [139]	Poor handling, Stress concentrators	The multi-layer composite pipe has the ultimate fundamental properties to deliver fuel and natural gas. The internal layer will undergo brittle fracture; polymers like PE, PP, etc. can provide resistance against the internal environment.	2018
Polymer crystallinity and the ductile to brittle transition [140]	Permeation (Immersion)	The PA-11 was aged in water and organic acids; the molecular weight degradation increases the crystallinity. Ductility decreased due to chemi-crystallization. The heat of fusion value determined the transition from ductile to brittle.	2018
Approaches for Safety Analysis of Gas Pipeline Functionality in Terms of Failure Occurrence: A Case Study [141]	Mechanical Damage (outside)	The failure rate of PE in gas distribution networks was given from the company in Poland is λp = 0.18 km^−1^ a^−1^. The percentage of failure (95.28%) is mainly supported by mechanical damage, including human activity, out of date plans, and building of other infrastructures.	2018
Innovative Field Trial Results of Flangleess Grooved HDPE Liner Application in a Super Gigantic Field for Oil Flow Line Internal Corrosion Management [142]	Permeation	ADNOC installed a 3-inch grooved HDPE liner in one of their onshore oil fields. After five years of service, testing was carried out, and the reduction in density and permeation of hydrocarbons was seen by GC-MS analysis	2018
Failure Analysis of Buried Polyethylene Pipe Subjected to Combined Loading of Non-uniform Settlement and Landslide Based on FEM [143]	Outside damage	The PE pipes used for urban gas transportation are more prone to landslide, settlement, and traffic load. The damage caused by a landslide is more prominent than settlement.	2018
Sustainable and safe in exploitation of gas networks. Part1. Stress factors of plastic pipelines [144]	Human error	The PE pipe used for the transportation of natural gas were frequently failed due to human error. These include the defective installation design, poor handling, and failure to comply with technical regulations.	2018
Thermal-oxidative aging performance and life prediction of polyethylene pipe under cyclic and constant internal pressure [145]	Thermal aging	The presence of carbonyl and hydroxyl groups on the surface-displayed thermal oxidation. The internal cyclic pressure broke the molecular chains rapidly and promoted degradation. The life of PE100 facing cyclic stress is 27.96% shorter than constant stress.	2019
Analysis of cracks in polyvinylidene fluoride lined reinforced thermoplastic pipe used in acidic gas fields [146]	Weld cracks	The PVDF liner was used for transporting oil and gas at 90 bar and 37 °C in the presence of acidic gases. The liner failed after six months due to weld lines formed after improper processing.	2019
Creep behavior of in-service flexible flowline polyamide 11 [81]	Permeation	The samples were taken from 3 different layers of PA11 flexible pipe after three years. The chain scission occurred, which in turn reduces the CIV value and elastic modulus.	2020
Development of Carbon Fiber Based Structural Health Monitoring System for Glass Fiber-Reinforced Polymer Composite Conduits [147]	Internal Pressure (Creep & Fatigue)	Leakage is mainly observed in internally pressurized pipes. The matrix cracking occurred due to an increase in static or cyclic loading. Due to the low strength of the matrix, delamination occurred.	2020
Damage evaluation and protection method of resin pipe for gas conduit subjected to impact load [148]	Third-party damage	The impact resistance of the PA pipe is better than HDPE and MDPE pipe. The MDPE pipe with protective sheets, including reinforced fiber and non-woven fiber provide better static and impact resistance than the HDPE pipe. In 2015 the US Department of Energy provided that the gas distribution sector contained mostly plastic pipes to cater corrosion.	2020

**Table A2:** Permeation (Exposure and Immersion).

Materials	Environment (Gases or Liquids)	Parameters	Permeation Outcomes	Ref
MDPE	CO_2,_ CH_4_, Aromatic hydrocarbon	T: ambient P: 100 psi Thick: 10 mm	MDPE swells up to 15% in the presence of hydrocarbons. CO_2_ has greater permeation in MDPE than CH_4_.	[149]
HDPE	CO_2_, CH_4_	T:40,60,81 °C P: 4 MPa Thick: 2 mm	Pe and S coefficients increase due to an increase in thickness. Pe and D coefficients increase as pressure increases. In the case of CO_2_, the Pe and D coefficients value increases as compared to S by increasing temperature.	[150]
PE80, PE-X, PE-DB (Al-foil)	33% H_2_S, 10% CO_2_, 70% CH_4_	T:50 °C P: 40 & 80 bar Thick: 5 mm Duration: 3 months	Permeation of acidic gases creates corrosive conditions in the annulus. The corrosion product layers were greater in the case of 80 bar.	[151]
PE-80	CO_2_, CH_4_, CO_2_/CH_4_, H_2_/CH_4_	T: 60 °C P: 40 bars Thickness: 1–3 mm	The CO_2_ molecules diffusivity is greater than CH_4_. In the case of a mixture of gases, no particular gas-gas and gas-polymer interaction were observed.	[152]
PE100 PA11	H_2_, CH_4_	T: 20,50,80 °C P: 5 & 20 bars Thick: 1 mm Duration: 13 months	No effect on the mechanical properties observed after the aging process. PE100 is more permeable to H_2_ than PA11. Pe coefficient is unaffected at 20 °C under pure H_2_.	[97]
PA (TP30)	CO_2_, H_2_S, CH_4_, Liquid (H_2_O)	T: 140 °C	Water has a more prominent effect as compared to gases.	[153]
PVDF	H_2_S	T: 100 °C P: 60–70 bars Duration:339 days	SOUR JIP investigated the performance of PVDF at MERL ltd. No effect was observed on the weight and mechanical properties of PVDF liner.	[42]
PE100, PA11, PAHM	H_2_, CH_4_	T: 20,50,80 °C P: 5 & 20 bars Thick: 1 mm Duration: 1 year	The degree of crystallinity is higher for HDPE than for others. Permeation rates for H_2_ are higher despite the temperature, pressure, and the mixture of gases.	[154]
HDPE, PTFE	H_2_	T: ambient P:100 MPa Duration: one week	No significant change was observed in Tg and tensile strength. HDPE has a lower permeability than PTFE due to higher crystallinity.	[155]
PA12, HDPE	12.5% H2S, 1.3% CO_2_, crude oil	T: 45 °C P: 290 psi Thick: 6.7 mm	The Pe coefficients of methane and light hydrocarbons in HDPE is higher as compared to PA12. Tensile modulus decreased while elongation at break increased.	[102]
PPS, PEEK	CO_2_, H_2_S	T: 80 & 100 °C P: 400 bars Thick: 2.3 mm	The Pe and D coefficients increase with temperature in the presence of CO_2_. The solubility of H_2_S is not prominent due to low partial pressure (2.25 bar).	[156]
TFE (PFA)	HCl, NH_4_	T: 25 °C Thick: 0.25 mm, round sample with 4.6 cm dia	Ammonia has a greater affinity for PFA. Ammonia has higher Pe and D coefficients as compared to HCl.	[157]
PVDF ECTFE	HCl, HBr	T: 70 °C & 80 °C Thick: 1.35–1.5 & 3–5 mm Duration: 24 h	The diffusion rate of HCl is higher in PVDF as compared to HBr. The diffusion rate can be found out by the sorption desorption measurements and indicator techniques.	[158]
PA12 (PVDF outer layer)	Ethanol	T: 110 °C Duration: 2400 h	Molar mass was reduced, and the inner surface of the layer dissolved due to aging. The pipe became stiffer due to the loss of plasticizer.	[159]
HDPE with PA barrier	Biofuels (E85, Biodiesel, B10)	T: 20–40 °C Thick: 1.4–1.6 mm Duration: 5 years.	In biofuel, tensile strength first decreased then increased due to thickness variation. Its vice versa happened in biodiesel. No noticeable change was observed in the FTIR spectra.	[160]
HDPE, PVDF	E10(CE10A), 55% butanol (CB55A) 10% Ethanol	Duration: 16 weeks.	10% volume change for PVDF and 16% for HDPE was observed. The samples show a reduction in % elongation.	[161]
HDPE	E30 (ethanol-gasoline) B30 (biodiesel)	T: 45 °C Duration: 1608 h	Mass and volume change was observed. Yield strength increases because of the absorbed fuel act as a plasticizer. Impact strength improves significantly due to the appearance of ductility after soaking.	[162]
HDPE	H_2_O	T: 23, 70, 90 °C	Mass gain increased with temperature. Ketones and carboxylic acids appeared in the FTIR spectra.	[163]
PE100 PE-RT	Brine, NORSOK M-710 (70% heptane, 20% cyclo-hexanes & 10% toluene)	T: 23,50,80 °C	Elastic modulus decreased, and its value is prominent at 80 °C. Oil saturation gives swelling, which affects the mechanical properties.	[164]
PVDF	CO_2_, CH_4_	T: 150 °C P: 300 bars	The diffusion and solubility coefficients have a linear relationship with temperature. Above 100 °C, the permeation of CO_2_ increased.	[165]
LDPE, PA11	Gases: CO_2_, CH_4_, H_2_O	T: Ambient P: 24 bars	The composite film provides impermeable performance due to the presence of the Al continuous layer. The mass gain due to corrosion products was higher in LDPE and nylon.	[88]
PE-80	Gases: CO_2_, CH_4_, H_2_S	T: 60 °C P: 2k bars Thick: 1–4 mm	The gas solubility got decreased at high pressures due to the hydrostatic effect. The volume of the amorphous phase decreased down to 6% at 2k bars due to less free volume available.	[166]
PA11	H_2_S	T:80 °C P: 200 bars	The anti-H_2_S layer stopped the permeation due to the presence of PEZnO. This zinc oxide has higher reactivity with H_2_S, proved by a full-scale flowline qualification test.	[167]
PA11 PA12	CO_2_, H_2_O, Crude oil	T: 80,100,120 °C P: 20 bars	The CIV value of PA11 was lower than PA12 after aging. PA12 has good % elongation.	[168]
HDPE	CO_2_, water, and sand		The abrasive wear resistance of HDPE was 3.2 times better than CS. After the 12 months field trial, no swelling or softening in HDPE was observed.	[169]
HDPE	Aromatic hydrocarbons, Crude oil, diesel		Loss of mechanical properties occurred due to swelling and plasticization. Volume increase up to 10% for aromatics and less than 10% for crude oil, diesel, and TM mixture (toluene-methanol)	[95]
HDPE	Methanol, 1-butanol		Toluene has a more aggressive impact on HDPE than methanol. It took around one week to reach equilibrium as compared to 1-butanol.	[170]
HDPE, XLPE	crude oil, toluene, cyclohexane, and n-heptane		The volume fractions were almost the same for both excluding toluene. The value of young’s modulus and the yield stress affected the swollen samples due to the plasticization of the amorphous components	[171]
PVDF	Amines, esters, Ketones		Its water absorption is almost equal to 0.04 within 24 h. Strong bases, amines, esters, and ketones cause swelling, softening, and dissolution	[43]
